# Challenges for the registration of vaccines in emerging countries: Differences in dossier requirements, application and evaluation processes^☆^

**DOI:** 10.1016/j.vaccine.2018.03.049

**Published:** 2018-06-07

**Authors:** Nora Dellepiane, Sonia Pagliusi, Registration Experts Working Group

**Affiliations:** a QRB Consultants, Spain; b DCVMN International, Switzerland

**Keywords:** Vaccine, Regulatory convergence, Marketing authorisation, Common Technical Document, Emerging countries

## Abstract

The divergence of regulatory requirements and processes in developing and emerging countries contributes to hamper vaccines’ registration, and therefore delay access to high-quality, safe and efficacious vaccines for their respective populations. This report focuses on providing insights on the heterogeneity of registration requirements in terms of numbering structure and overall content of dossiers for marketing authorisation applications for vaccines in different areas of the world. While it also illustrates the divergence of regulatory processes in general, as well as the need to avoid redundant reviews, it does not claim to provide a comprehensive view of all processes nor existing facilitating mechanisms, nor is it intended to touch upon the differences in assessments made by different regulatory authorities. This report describes the work analysed by regulatory experts from vaccine manufacturing companies during a meeting held in Geneva in May 2017, in identifying and quantifying differences in the requirements for vaccine registration in three aspects for comparison: the dossier numbering structure and contents, the application forms, and the evaluation procedures, in different countries and regions. The Module 1 of the Common Technical Document (CTD) of 10 countries were compared. Modules 2–5 of the CTDs of two regions and three countries were compared to the CTD of the US FDA. The application forms of eight countries were compared and the registration procedures of 134 importing countries were compared as well. The analysis indicates a high degree of divergence in numbering structure and content requirements. Possible interventions that would lead to significant improvements in registration efficiency include alignment in CTD numbering structure, a standardised model-application form, and better convergence of evaluation procedures.

## 1 Introduction

The United Nations’ System for vaccine procurement and supply is served by the United Nations International Children's Fund (UNICEF) and the Pan-American Health Organisation revolving fund (PAHO-RF). It relies on the World Health Organisation prequalification programme (WHO-PQ) to pre-select vaccines eligible for purchase as well as to monitor the quality, safety and efficacy of the vaccines supplied to receiving countries [1], [2]. The UN system targets low middle income (LMIC) and low-income countries (LIC). Vaccines procured through this centralized system to support National Immunisation Programmes, have to fulfil three requirements: a valid marketing authorisation, evaluation by the WHO prequalification programme and, in some cases, marketing authorisation evaluation in the receiving countries.

Although these three levels of authorisation are required, the dossier review process should not need to be repeated at each level. Ideally, a vaccine that is well regulated in the manufacturing country and is prequalified by WHO, fulfils in principle the requirements of safety, efficacy and quality, and should be eligible for an accelerated and facilitated process for marketing authorisation in the receiving countries, based on recognition of the dossier evaluations performed by the manufacturing country competent NRA and the WHO. Although the WHO has developed and promotes a collaborative registration procedure for generic pharmaceuticals with the receiving countries’ NRAs, recently extended in principle to vaccines [3], due to the need for adaptations, advocacy and intensive mentoring by WHO, which requires significant efforts and resources, its level of implementation remains low for vaccines.

Practically, this means that the manufacturers applying for registration of WHO prequalified vaccines undergo a similar process twice, and a third time in each individual country, being subject to different national requirements, in receiving countries. This repetitive registration process implies high number of dossiers prepared for one and the same vaccine, adding little value to the licensed products and delaying vaccine access for some populations.

There have been numerous attempts to align regulatory requirements between countries and regions, as well as attempts encouraging mutual recognition practices between regulators of different countries in order to save both resources and time, avoiding redundancy. One such international initiative is represented by the International Council for Harmonisation (ICH) of technical requirements for pharmaceuticals for human use, originally established by the European Union, Japan and the United States of America in 1990 and expanded to other member and observer countries [4]. The ICH developed and promoted the use of a Common Technical Document (CTD) which represents a common dossier for regulatory submissions for use in the ICH countries [5]. The CTD has subsequently been adopted by additional countries globally, which should have led to a harmonisation of requirements. Countries adopting the CTD have however made local individual adaptations of the ICH CTD template, thus defeating the original intention of harmonisation. Hence, the divergence of requirements between countries remains high and evident in two-areas: (a) dossier numbering structure and contents and (b) the registration application/evaluation procedure.

The existing divergence in content requirements and registration procedures seriously impact the timelines for registration, because manufacturers are required to comply with a diversity of country specific requirements and because the NRAs have different times for evaluation of the submitted information. This results in lengthy processes delaying unnecessarily the access to high-quality, safe and efficacious vaccines in developing countries.

The lack of awareness of the magnitude of the divergence in dossier requirements and regulatory approval procedures is such that vaccine manufacturers have considered it important to invest some effort and resources to analyse these differences. This paper describes the results of a systematic comparison of CTD numbering structure and contents, based on available guidelines from selected countries, showing the similarities and differences in the requirements. It also describes the application and evaluation procedures for registration experienced in different countries, highlighting the magnitude of the problem, as well as identifying opportunities for improvements in alignment.

## 2 Working methodology

The Developing Countries Vaccine Manufacturers’ Network (DCVMN) [6], commissioned a comparative analysis of the CTD requirements in different countries in order to estimate the similarities and differences for the different CTD modules. The results of this work were presented to a group of registration experts from DCVMN and IFPMA affiliated vaccine manufacturers, in an informal workshop held in Geneva on 15 and 16 May 2017 [7], where the participants (a) reviewed the outcome of the comparative analysis for each of the CTD modules and made corrections and adjustments, (b) listed the procedural differences between 134 countries worldwide and (c) compared the application forms required by different countries.^2^

According to the ICH, the CTD includes 5 modules. Module 1 is not harmonised and contains regional/country information. Each country or region has its own numbering system and requirements [8]. Modules 2–5 are harmonised modules, and include information regarding quality, safety and efficacy.

To assess similarities and differences between countries’ CTD structures, and in order to have representation across the globe, the following regions/countries technical dossiers were included in the comparison: Australia [9], the Association of Southeast Asian Nations (ASEAN) [10], China [11], the European Union [12], the Gulf Cooperation Council (GCC) [13], India [14], Jordan [15], the Pan American Health Organisation (PAHO) [16], the United States of America Food and Drug Administration (FDA) [17], [18], [15], Tanzania [19] and Thailand [20]. The WHO prequalification programme (WHO-PQ), has recently decided to adopt the CTD structure for the prequalification submissions, and has proposed requirements for Module 1 which were published for public comments [21]. This was also included in this comparison. The Module 1 of these countries or regions were compared to each other to assess similarities and differences. For simplicity of the comparative analysis, item 1.2 (application forms) was left out.

Assuming that Modules 2–5 are harmonised modules, it was decided to include fewer countries in the comparison of these modules. It included the ICH CTD and those proposed by two regions of the world (ASEAN and PAHO) in addition to India, as a major vaccine exporting country, Jordan, representative of countries in the Eastern Mediterranean region and Thailand (currently does not follow fully the ASEAN CTD). Each of these CTDs were compared against the ICH as implemented by the US FDA and similarities and differences evaluated.

For the analysis of Module 1, contents expressed exactly in the same terms or requiring the same information were considered “similar”; and contents that differed between the CTDs were considered “different”. For the analysis of modules 2–5, requirements from different countries were considered “different” from the ICH CTD if one of the following situations applied:

(1) Country X does not require specific items required in the ICH CTD
(2) Country X requires information not required in the ICH CTD (other information)
(3) Country X contains in its requirements similar heading as in the ICH CTD but the information required under such heading is not specified, while specified in the ICH CTD.
(4) Country X contains in its requirements similar heading as in the ICH CTD but the information required under such heading is specified, while not specified in the ICH CTD
(5) Country X requires different information from ICH under the same heading
(6) Country X requires different information from ICH under the same numbering

The structure of the ASEAN CTD is different from the ICH CTD. Information required in Module 2 of the ICH CTD is embedded in other sections in the ASEAN CTD. Due to these structural differences, the comparison between the ICH and the ASEAN CTD was done separately from the other countries.

Percentages of similarity were calculated using the following formula for Module 1 and Modules 2–5 respectively.

Module 1

$$\%\;\text{of similarity}=\frac{\text{No. items with similar content or numbering}}{\text{No. items compared}}\times 100$$

Modules 2–5

$$\%\;\text{of similarity}=\frac{\text{No. items with similar content or numbering to ICH}(\text{CTD})}{\text{No. items compared}}\times 100$$

%of difference=100-%of similarity

Furthermore, the meeting participants analysed and compared the application forms (item 1.2 not considered in the comparison of Module 1) from eight countries (Cuba, Egypt, EU, Indonesia, Iran, Iraq, Jordan, and United Arab Emirates) aiming at identifying the critical information included in the majority of them.

The registration procedures and requirements in different countries were also identified with a total of a hundred and thirty-four countries included in the analysis.^3^

## 3 Comparison of CTD contents and numbering between countries

To compare Module 1, items were organised in tabular form: topics with similar content were aligned in the same row independently of the section numbering used in the different CTDs. The comparison was based on similarities or differences, both in terms of contents and numbering. Table 1 shows an excerpt of this comparison for some of the items contained in Module 1. For example, the first item on the table refers to mock-up labelling which is required in six of the ten countries as well as in the WHO proposed Module 1. However, the numbering for this topic differs between the individual countries as well as for WHO.

**Table 1:** Excerpt of comparison of similarities and differences of the contents as appearing in Module 1 of CTDs from Australia, China, Europe, GCC, India, Jordan, PAHO, Tanzania, Thailand, USA and WHO-PQ.

**Fig. 1:**
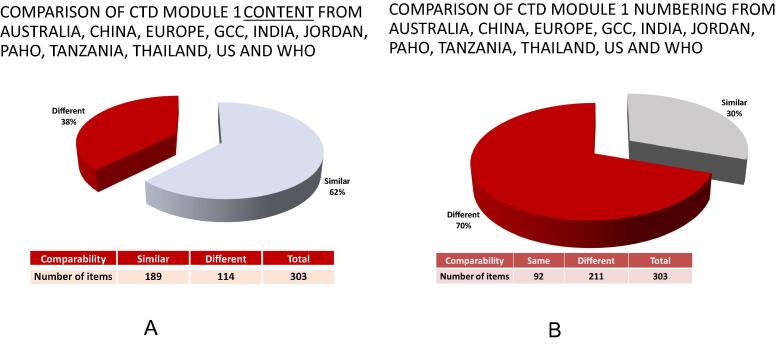
Comparison of CTD Module 1 across 10 countries. This figure shows the comparison of Module 1 of CTDs from Australia, China, Europe, GCC, India, Jordan, PAHO, Tanzania, Thailand, USA and WHO-PQ proposed Module 1. (A) The results of the comparison related to the contents of headings/subheadings, and (B) the results of the comparison related to the numbering of heading/subheadings. The pie charts show the percentage of similarity and difference. The percentage of differences is indicated in red color. The data in the table under the pie charts show the number of items compared and how many of those were either similar or different, both in contents and numbering. (For interpretation of the references to color in this figure legend, the reader is referred to the web version of this article.)

Fig. 1 shows that for Module 1, for which 303 items were compared for content (Fig. 1A), the overall level of similarity for all the CTDs included in the study was 62%. The level of similarity observed when the numbering was compared (Fig. 1B) was only 30%.

Table 2 provides an example for part of Module 2 of the way in which the content of the CTDs was summarised to facilitate the comparative work. Highlights in light blue indicate items that are similar to the ICH CTD templates and those highlighted in red show the items that are different.

**Table 2:** Excerpt of comparison of similarities (blue) and differences (reddish) in the contents (heading and sub-headings) of Module 2 as appearing in the CTDs from PAHO, India, Jordan, ASEAN and Thailand, when compared to the ICH CTD (FDA) for reference (white column).

While the comparison between the ASEAN and the ICH CTDs showed 93% of similarity for content, it gave 100% difference in numbering, due to the difference in dossier structures. Table 3A shows the results for contents obtained when the Modules 2–5 from PAHO, India, Jordan and Thailand CTDs were compared to the ICH CTD (FDA). Table 3B shows the results for numbering. The overall results indicate 23% similarity in content and 21% similarity for numbering.

**Table 3:** Quantitative analysis of overall similarities and differences in Modules 2–5 from CTDs from PAHO, India, Jordan, ASEAN and Thailand as compared to the ICH (FDA) CTD. A: analysis of contents (headings & sub-headings). B: analysis of numbering.

## 4 Comparison of vaccine registration procedures in 134 countries

**Fig. 2:**
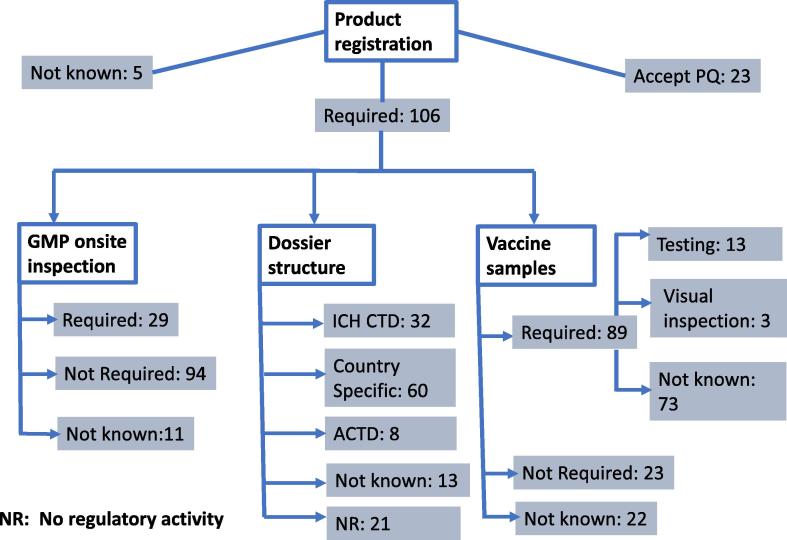
Evaluation processes for vaccine registration in 134 countries. The figure shows the analysis of 134 countries, classified as those that require registration of WHO prequalified vaccines (n = 106), those that accept the prequalification without further requirements for registration (n = 23) and those where the requirements are not known (n = 5). From the 106 that require registration, it shows the break down of the number of countries that require GMP onsite inspection of the facilities, or do not require or have an unclear status in relation to a GMP onsite inspection. Furthermore, the format of dossier and requirement of samples were categorised. From those countries that require samples it shows whether it is for testing purposes, for visual inspection or with unclear purposes. The data collected is based on practical registration experiences only, at specific time points, thus it is indicative in nature.

The marketing authorisation evaluation process in ICH member countries also differs, based on country guidelines. For instance, the USA bases its assessment on a CTD review only (unless new facilities and/or new manufacturing process are involved), the EU bases its assessment on the review of the CTD and GMP onsite inspection as needed; Japan requires previous license of the facilities, while Canada requires licensing of the establishment, an onsite evaluation (for Biologics only) and testing of batches. Due to the differences in the evaluation process between these ICH member countries, the experts considered it relevant to assess the magnitude of divergence in the evaluation process at global level, with emphasis on countries supplied through the United Nations centralised procurement system. Fig. 2 illustrates the procedures in 134 countries. Out of the countries analysed, only 29 required an onsite inspection to be conducted for approval (licensing) of the manufacturing facilities as a pre-condition for marketing authorisation submissions. The situation was unclear for 11 countries and the remaining 94 did not require prior onsite inspection for licensing of the manufacturing facilities. Fig. 2 summarises the results observed with regards to vaccine registration in 134 countries.

Twenty-nine countries conduct a GMP onsite inspection as part of the evaluation process, independently of the number and quality of onsite inspections previously conducted by other regulatory bodies of the same facilities. The figure also reflects the variability in dossier structures used, with 60 countries having a specific dossier.

Twenty-three countries accept WHO prequalification as basis for local registration, and twenty-one countries were identified as not having any regulatory activities (Fig. 2). Eighty-nine countries require vaccine samples as part of the registration evaluation process; the purpose of such requirement is unclear for 73 of these countries.

In addition to the overall steps of the registration procedure, 8 countries were identified that require the performance of local clinical trials in order to accept a registration submission. Some of these countries have provisions for waivers under special circumstances or on a case-by-case basis.

Additional country specific requirements further complicate the regulatory process. For example, many countries require the Certificate of Pharmaceutical Product (CPP) issued by the NRA responsible for the regulatory oversight of the vaccine, and in addition, some countries require prior approval in “reference countries”. Reference countries are considered to have stringent/robust regulatory systems and hence prior approval in these countries represents a “quality label”. Usually, countries applying such prior approval requirement list the reference countries that are considered acceptable. The requirement may be limited to marketing authorisation in the reference country or include the requirement for actual commercialisation in the reference country. This represents an additional challenge, particularly if the vaccine in question is not needed in the manufacturing nor in a reference country.

Furthermore, labelling and packaging requirements differ between countries, in terms of contents and language. Container labels are normally required to be printed in the local language.

In view of the above variability in evaluation processes and country specific requirements, the timelines for registration differ significantly as well. Many countries in Central and East Africa need an average of 24 months for registration, while most countries in West Africa need between 6 and 12 months for registration but require prior approval in France or EU. Many countries in the Middle East follow a quicker registration process, if the product has been pre-approved in Saudi Arabia.

A study published by Ahonkhai et al. reports that the time between the first and last registration of 8 vaccines in 20 countries of Sub Saharan Africa spanned a medium of 78 months and the time span for the registration of a new drug was also lengthy, with a median of 52 months [22].

## 5 Comparison of application forms

Application forms are usually included in Module 1 and are required in some instances in advance of the dossier submission. The working group decided to analyse the specific information required in application forms (separately from Module 1).

Table 4 shows five main categories for the information required as part of the application form. Application forms from eight countries (Cuba, Egypt, EU, Indonesia, Iran, Iraq, Jordan and UAE) were analysed and the required information grouped into the categories listed. A sixth group was added for additional information required in some countries. For each topic under each category the table shows the number of countries requiring the specified information. The order in which the information is required and the numbering used in the forms is country specific, which complicates the possibility of alignment of the information.

**Table 4:** Number of countries requiring specific information in application forms across 8 countries. The specific information required on the application form of 8 illustrative countries, is listed in the text of columns, arranged under 6 major categories, indicated in the upper row (light grey). The number of countries requiring each specific information is shown next to each row, describing the content of the respective information. MAH = marketing authorisation holder.

Eighteen items are required in relation to the “Information about the product”. For this specific group, the requirements are slightly more homogeneous and items required by a few countries (2 or 3) are considered less relevant for inclusion in the application form. For example, the type and sources of strains used, vaccine antigen master file, active substance master file and clinical trial summary among others, are part of the technical modules of the dossier. It is often unclear why such information is required in the application form. The information regarding the regulatory status of the product is very important; however, some of the items are required both in Module 1 and in the application form, thus redundant for some of the items. Nine items are considered under the category “Information about the applicant and distributor”. The number of countries requiring such information varies between two and eight. It is surprising to see that the company name is required in 6 countries and similarly for the name of the marketing authorisation holder when such information is key and should be required by all countries. The five items required under “Storage conditions and shelf life” are relevant and reflected by the majority of countries demanding them. The “Labelling, packaging and insert information” is part of Module 1 of the CTD and requiring this information again in the application form may be redundant. Additional information required by some countries includes the pharmacovigilance system, which is also part of Module 1; pricing details (required by half of the countries) in addition to specific legal documents. Patent information is included, however none of the countries considered in this analysis required such information.

## 6 Discussion

This report addresses three aspects of the processes involved in the evaluation of vaccines for registration/marketing authorisation globally: the numbering structure and contents of CTDs used in different countries; the evaluation process with the specific country requirements; and the information required in the application forms. For the latter, 8 countries’ forms were reviewed.

A caveat in the analysis is that the ICH CTD is applicable to both medicines and biological products, while the PAHO, Thailand and Jordan CTDs are specific for vaccines and biological products only. This is a source of discrepancy that cannot be avoided. However, the heterogeneity observed in contents and numbering of the CTD templates reviewed was significant.

Meeting participants agreed that the degree of difference was higher for Modules 2–5 of CTDs (more than 75% different in contents and above 79% different in sections’ numbering) than for Module 1 (38% different in content and 70% different in numbering), which contradicts the fact that Module 1 is non-harmonised and expected to include “regional/local information” while the other four modules are supposedly harmonised. Discrepancies in the terminology were also observed. The addition or deletion of information means that the numbering used is not harmonised across countries leading to having the same numbering for information that is different and unrelated, and different numbering for similar information. The adoption of the CTD by an increasing number of countries is welcome; however the challenge remains in the “adaptations” made to the ICH CTD to fit the demands of each country.

A paper recently published by Pombo et al. [23] describes the number of countries in the region of the Americas that have adopted the PANDRH Technical Document No.1 (TDN01), providing the guidance for the development of the CTD to be used in the region. The paper concludes that 15 countries have specific requirements for the registration of vaccines, most of them convergent with TDN01. However, a detailed analysis of contents, structure of the respective CTDs shows that most of them differ at least in numbering structure, while others differ in contents as well. Such results highlight the need for further alignment: the divergence in numbering and structure is more important than may seem at first sight. Even if the technical information required (content) is reasonably aligned, the need to present such information in different dossier structure, according to the local required numbering, is time and resources consuming.

Consequently, the existing divergence between regulatory requirements and registration procedures worldwide forces the regulatory groups of manufacturing companies to prepare a tailor made CTD for each country where they apply for registration. This means practically reworking the same information to meet different formats with no or very little added value. Overall this leads to redundant efforts and lengthy regulatory processes and delayed access to these much-needed vaccines for the target population.

The experts believe that there is opportunity for a higher level of alignment. For example, countries adopting the CTD requiring more detailed information under certain items, could sub-itemize the information under a single heading. Conversely, if certain information is not required, the relevant heading and the section number could be omitted. Such a simple means of maintaining a harmonised numbering system would have a positive impact in the efficiency of vaccine registration files for submission, in terms of time and resources.

Attempting higher level of convergence in the evaluation process is feasible, while Harmonisation would appear to be a more challenging and lengthy task. According to the principles of good regulatory practices consideration should be given to “establishing regulations with sufficient flexibility to allow for participation in international cooperation frameworks, such as for information-sharing, convergence, harmonisation, work-sharing, reliance and recognition” [24]. UN procured vaccines are usually WHO prequalified. As such, they are firstly granted a marketing authorisation by the NRA responsible for the regulatory oversight of the vaccine in the manufacturing country and subsequently undergo the prequalification evaluation, focusing on the quality, safety, efficacy and the programmatic suitability of the vaccines for use in the national immunisation programmes in LMICs. Having met the expectations of the supervisory National Regulatory Agency in the manufacturing country and those of the WHO, such vaccines should be subject to an expedited and facilitated review procedure by the regulatory authorities in the receiving country based on reports available from the two prior evaluations performed.

Experts discussed that two key concepts have been advocated for years but still fail to be implemented by all countries; these are the concept of reliance/recognition of regulatory reports and that of avoidance of redundancy, or repetition of testing and inspections. Reliance on work performed/reported by other regulatory bodies or international agencies can be achieved through information sharing agreements or mutual recognition. Redundant testing and inspections could also be avoided with similar arrangements. Available mechanisms to address this problem include the collaborative procedure promoted by WHO, which bases the registration in the receiving countries on information sharing between WHO and the receiving country NRA, bilateral agreements between countries and regional agreements based on economic blocks collaborations. Some of these mechanisms are more effective than others, however the degree of divergence still remains high. An integrated framework for regulators, Ministry of Health and procurement agencies is much needed for NRA convergence to progress.

The third area highlighted by the workshop participants was the analysis of application forms from 8 countries. The comparison of documentation required by these countries showed that there is some consensus on which documentation is considered important. However some documents are required only by a few countries (e.g. vaccine antigen master file). Such information, could be captured elsewhere in the dossier. Additionally, there is a third group of documents required by some countries in their application forms which is also provided in other modules or sections of the CTD. The comparative analysis of the application forms shows, once more, that there is scope for higher level of alignment through the development of a model-standardised application form. Such a model application-form would capture the relevant information to be provided and avoid inclusion of data already provided in other modules or sections of the CTD, such as clinical and toxicological data. This could be based on an agreed common numbering structure and similar information would be sub-categorised under one heading. A proposal for a “standardised” application form will be prepared and circulated for consideration by regulators and regulatory networks, including WHO, ICH, ASEAN, AVAREF, PANDRH and others who can best support the implementation of alignment initiatives and mutual recognition agreements among regulatory authorities, to improve access to life-saving vaccines, while reducing time and streamlining resources.

## 7 Conclusions

The analysis undertaken by the global registration experts highlighted the current divergence of regulatory requirements for registration of vaccines worldwide, based on publicly available data, and points to the need for convergence initiatives. The group felt the impelling need to share this information with the immunisation community including groups of regulators, programme managers, procurement agencies, donors and other partners. Increased understanding and awareness of the specific challenges may help NRAs and regionally based stakeholders to consider practical initiatives to reinforce recognition and avoid redundancies, while identifying potential solutions. Manufacturers have also, through this analysis, identified some of the possible interventions that would lead to significant improvements. These include proposals for alignment in CTD numbering structure, a standardised template for application forms, as well as better convergence of file content and evaluation procedures. It was agreed that vaccine registration processes should be streamlined and redundancies removed towards enabling faster access to vaccines in developing countries.

## Collaboration authors

Mary Allin (Pfizer, UK), Abdulaziz Almutairi (Arabio, Saudi Arabia), Paula Barbosa (International Federation of Pharmaceutical Manufacturers and Associations, Switzerland), Nirav Amitkumar Chokshi (Zydus Cadila, India), Monique Collaço de Moraes Stávale (Bio-Manguinhos, Brazil), Samir Desai (Zydus Cadila, India), Shubhangi Ghadge (Serum Institute of India, India), Tarek Ibrahim (Arabio, Saudi Arabia), Seon Gyeong Jeong (LG Chem, South Korea), Matthew Marsden (Pfizer, UK), Mic McGoldrick (Merck Sharp & Dohme, USA), Yijie Qu (China National Biotech Group, China), Christophe Saillez (former GSK, Belgium), Mira Utom (Biofarma, Indonesia), Qiaoruo Xiong (China National Biotech Group, China).

## Footnotes

2 Participants in the workshop were regulatory experts either from companies with WHO prequalified vaccines or registration experienced at global level.

3 Countries included in the study analysis: Albania, Algeria, Angola, Armenia, Argentina, Azerbaijan, Bahrain, Barbados, Bangladesh, Belarus, Benin, Bhutan, Bolivia, Botswana, Brazil, Burkina Faso, Burundi, Cambodia, Cameroon, Cape Verde, Central African Republic, Chad, Chile, Colombia, Congo Brazzaville, Cook Islands, Costa Rica, Cuba, Democratic Republic of Congo, Dominican Republic, Democratic Peoples’ Republic of Korea, Ecuador, Egypt, El Salvador, Eritrea, Equatorial Guinea, Ethiopia, Fiji, Gabon, Gambia, Golf Countries Community (GCC) States, Georgia, Ghana, Guatemala, Guinea Bissau, Guinea Conakry, Guyana, Haiti, Honduras, Hongkong, Indonesia, Iran, Iraq, Ivory Coast, Jamaica, Jordan, Kazakhstan, Kenya, Kiribati, Kuwait, Kyrgyzstan, Lao PDR, Lebanon, Lesotho, Liberia, Libya, Macedonia, Madagascar, Malawi, Malaysia, Maldives, Mali, Mauritania, Mauritius, Mexico, Moldova, Mongolia, Morocco, Mozambique, Myanmar, Namibia, Nauru, Nepal, Nicaragua, Niger, Nigeria, Oman, Pakistan, Palestine, Panama, Papua New Guinea, Paraguay, Peru, Philippines, Qatar, Republic of Korea, Republic of Togo, Republic of Yemen, Russia, Rwanda, Samoa, Santo Tome, Senegal, Serbia, Seychelles, Sierra Leone, Solomon Islands, South Africa, North Sudan, South Sudan, Suriname, Swaziland, Syria, Tajikistan, Tanzania, Thailand, Timor-Leste, Trinidad & Tobago, Tokelau, Tonga, Tunisia, Turkey, Turkmenistan, Tuvalu, Uganda, Ukraine, United Arab Emirates, Uruguay, Uzbekistan, Vanuatu, Venezuela, Vietnam, Zambia, Zimbabwe.

## References

[1] World Health Organization. Procedure for assessing the acceptability, in principle, of vaccines for purchase by United Nations agencies. WHO Technical Report Series 978, Annex 6; 2013.

[2] Dellepiane N., Wood D. (2015). Twenty-five years of WHO vaccines prequalification
programme (1987–2012). Lessons learned and future
perspectives. Vaccine.

[3] Collaborative procedure between the World Health Organization (WHO) Prequalification Team and national regulatory authorities in the assessment and accelerated national registration of WHO-prequalified pharmaceutical products and vaccines. WHO Technical Report Series 996, Annex 8; 2016.

[4] ICH membership. Current members and observers http://www.ich.org/about/membership.htm [accessed 02.06.17].

[5] ICH Harmonised Guidelines. Organisation of the Common Technical Document for the Registration of Pharmaceuticals for human use. M4. Current step 4; June 15, 2016.

[6] DCVMN. http://www.dcvmn.org/ [accessed 02.06.17].

[7] IFPMA: International Federation of Pharmaceutical Manufacturers and Associations https://www.ifpma.org/ [accessed 02.06.17].

[8] ICH CTD http://www.ich.org/products/ctd.html [accessed 02.06.17].

[9] CTD module 1. Administrative and prescribing information for Australia. Applicable to applications received by the TGA Version 3.0; 1st July 2015.

[10] The ASEAN Common Technical Document Dossier for the Registration of Pharmaceuticals for Human use. http://asean.org/storage/2017/03/68.-December-2016-ACTD.pdf [accessed 02.06.17].

[11] China Food and Drug Administration, Verification and issuance of registration certificates for imported chemicals (incl. from Hong Kong, Macao and Taiwan). 06 December 2013 http://eng.sfda.gov.cn/WS03/CL0769/98137.html.

[12] EU Module 1 eCTD Specification. Version 3.0 1; May 2016

[13] GCC Data Requirements for Human Drugs Submission http://sgh.org.sa/Portals/0/PDF/Cen_registration/GCC%20Data%20Requirements%20for%20Human%20Drugs%20Submission%20version%201.1.pdf [accessed 02.10.17].

[14] Indian CDSCO Guidance for Industry. http://www.cdsco.nic.in/writereaddata/CDSCO-GuidanceForIndustry.pdf [accessed 02.06.17].

[15] Jordan Food and Drug Administration. Registration requirements for pharmaceutical finished products according to CTD format http://www.nobles.com.jo/pdf/pharmaceutical/product/A-_reg._req._of_the_pharmacutical_products.pdf [accessed 02.06.17].

[16] PANDRH Network Technical Document No1. Harmonized requirements for the licensing of vaccines in the Americas and guidelines for preparation of application http://www2.paho.org/hq/index.php?option=com_docman&task=doc_details&gid=14516&Itemid=270&lang=en [accessed 02.06.17].

[17] FDA. The comprehensive table of contents, headings and hierarchy https://www.fda.gov/downloads/drugs/ucm163175.pdf [accessed 02.06.17].

[18] FDA. CTD overall table of contents (template) https://www.fda.gov/downloads/drugs/ucm163175.pdf [accessed on 17 October May 2017].

[19] Guidelines on submission of documentation for renewal of registration of human and veterinary pharmaceuticals products. August 2017. Available at Tanzania FDA website under https://www.tfda.go.tz/index/?q=medicines_downloads.

[20] The eCTD specification. Module 1 and regional information. Available at https://www.ipqpubs.com/wp-content/uploads/2014/10/TH-Module-1-and-Regional-Specification.pdf.

[21] WHO New format of Vaccine Prequalification dossier replaces the Product Summary File http://www.who.int/immunization_standards/vaccine_quality/Vx_PQ-Dossier/en/ [accessed 15.05.17].

[22] Ahonkhai V., Martins S. (2016). Speeding access to vaccines and medicines in low- and
middle-income countries: a case for change and a framework for
optimized product market authorization. PLOS ONE.

[23] Pombo M.L., Porrás A. (2016). Regulatory convergence and harmonization: barriers to
effective use and adoption of standards. Rev. Panam. Salud Pública.

[24] Good Regulatory Practices: Guideline for National Regulatory Authorities for Medical Products. WHO/DRAFT/ September 2016.

